# Treatment adequacy for social anxiety disorder in primary care patients

**DOI:** 10.1371/journal.pone.0206357

**Published:** 2018-11-05

**Authors:** Alexandra Chapdelaine, Jean-Daniel Carrier, Louise Fournier, Arnaud Duhoux, Pasquale Roberge

**Affiliations:** 1 PRIMUS Research Group, Faculty of Medicine and Health Sciences, Université de Sherbrooke, Québec, Canada; 2 Department of Psychiatry, Université de Sherbrooke, Québec, Canada; 3 Research Centre of the Centre Hospitalier de l’Université de Montréal (CRCHUM), School of Public Health, Université de Montréal, Québec, Canada; 4 Faculty of Nursing, Université de Montréal, Québec, Canada; 5 Department of Family Medicine and Emergency Medicine, Université de Sherbrooke, Québec, Canada; 6 Research Centre of the Centre Hospitalier de l’Université de Sherbrooke (CRCHUS), Québec, Canada; Department of Psychiatry and Neuropsychology, Maastricht University Medical Center, NETHERLANDS

## Abstract

**Objectives:**

There is a gap between clinical practice guidelines for social anxiety disorder and clinical practice that needs to be addressed to ensure the delivery of evidence-based treatments. The objectives of this study were: 1) to describe mental health service utilization in a cohort of primary care patients with social anxiety disorder; 2) to examine treatment adequacy for pharmacotherapy and psychotherapy according to indicators based on clinical practice guidelines; and 3) to explore correlates of treatment adequacy.

**Method:**

The “Dialogue” project (Quebec, Canada) is a large study conducted in 67 primary care clinics. After a mental health screening in primary care (n = 14 833), participants with anxiety or depressive symptoms took part in a telephone/web structured interview on mental health symptoms and service utilization (n = 1956). This study included 289 participants meeting DSM-IV criteria for social anxiety disorder.

**Results:**

Overall, 86.2% of participants reported consulting for mental health reasons over the past 12 months. Only 23.6% of our sample reported the detection of social anxiety disorder by a healthcare professional in the past 12 months. Approximately 2 in 5 respondents with social anxiety disorder reported receiving pharmacotherapy or psychotherapy meeting our treatment adequacy indicators. Antidepressant medication was the most common treatment. Logistic regression models showed that the detection of major depression (OR = 4.651; 95% CI: 2.559–8.453) or other anxiety disorder(s) (OR = 2.957; 95% CI: 1.555–5.625) were associated with receiving any adequate treatment, but the detection of social anxiety disorder itself was not (OR = 1.420; 95% CI: 0.696–2.899).

**Conclusion:**

Low rates of detection and treatment adequacy based on our indicators demonstrate that efforts must be made to ensure the quality of care for individuals with social anxiety disorder in primary care.

## Introduction

In Canada, the 12-month prevalence of social anxiety disorder (SAD) is estimated to be 3.2%, whereas lifetime prevalence is approximately 8.1% [1]. Individuals living with SAD experience intense discomfort when interacting socially due to the fear of acting in a way that could be negatively evaluated and/or lead to humiliation or rejection [2]. SAD is associated with avoidance behaviour [2] and is known to impair social functioning and to have negative consequences on educational attainment, occupational performance, relationships and quality of life [3–5].

For the treatment of SAD, whereas some clinical practice guidelines (CPGs) emphasize psychotherapy as the initial treatment when congruent with patient preferences [6], the Canadian Psychiatric Association considers either psychotherapy or pharmacotherapy as a first-line treatment [7]. The combination of pharmacotherapy and psychotherapy is not formally recommended in the latter CPGs [7] while NICE considers this option adequate for non- or partially responsive patients [6]. Cognitive behavioural therapy (CBT) is the gold-standard non-pharmacological treatment of SAD [6,7]. Regarding medication, first-line options include some SSRIs, venlafaxine and pregabalin, with second-line alternatives adding some benzodiazepines, gabapentin and phenelzine as reasonable treatment options [7]. Despite the availability of well-established CPGs, the treatment adequacy rates reported in previous studies are generally low, ranging from 34.1% to 38.7% for SAD [8–10], and from 27.0% to 54.5% for other anxiety disorders [8–16]. These studies highlight the gap between treatment guidelines and current practice, as well as the limited data available to understand this gap for SAD. In order to improve treatment adequacy rates at the population level, it is necessary to expand our understanding of the factors involved with receiving evidence-based treatment for SAD.

This study aimed to assess mental health service utilization and correlates of treatment adequacy in a large sample of primary care patients suffering from SAD in Quebec, Canada. Our specific objectives were: 1) to assess mental health service utilization; 2) to evaluate the adequacy of pharmacological and psychological treatments, based on indicators derived from CPGs; and 3) to identify correlates of treatment adequacy by investigating the contribution of conceptually distinct predisposing factors, enabling factors and need for care factors based on Andersen’s Behavioural Model of Health Care [17].

## Methods

### Study setting, participants and data collection

**Fig 1:**
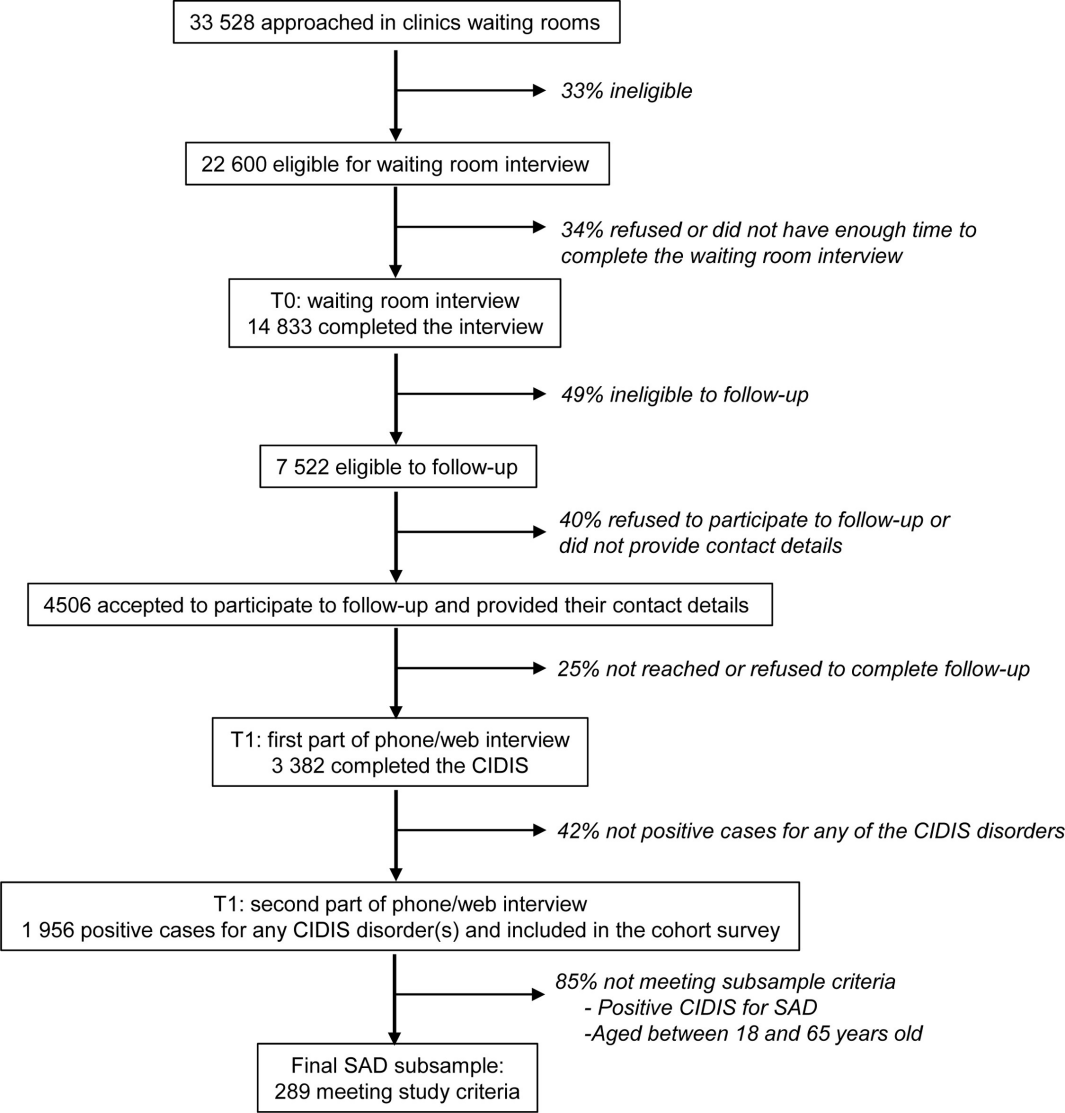
Dialogue project recruitment flowchart and subsample selection process.

This cross-sectional study used data drawn from the "Dialogue" project, a large cohort study initiated in 2008 which has been described elsewhere (see Roberge *et al*., 2015) [18]. Fig 1 presents the flowchart of the recruitment and selection process.

### Waiting room interview (T0)

Participants were first approached by a lay-interviewer in the waiting room of one of the 67 participating primary care clinics to fill a brief self-administered screening questionnaire including socio-demographic, overall health status, consultations with health care providers and psychotropic medication use questions, as well as the Hospital Anxiety and Depression Scale (HADS) [19,20]. Participants had to be 1) 18 years or older; 2) consulting a general practitioner for themselves; and 3) able to complete the questionnaire in French or English. A total of 14 833 participants completed this interview.

### Telephone/web interview (T1)

Waiting room interview participants were invited to a telephone/web follow-up interview if they reported either: elevated anxiety and/or depressive symptoms (≥8 [21,22] on the HADS [19,20]), any anxiety or depression medication use in the previous 12 months, anxious or depressive disorder diagnosis made by a physician, or consultation for mental health problems in the previous 12 months. The follow-up interview included the Composite International Diagnostic Interview–Simplified (CIDIS) [23], a brief structured psychiatric interview for lay-interviewers tailored to DSM-IV diagnostic criteria for common mental disorders [23]. Participants were also asked questions on experience of care, service utilization for emotional reasons, medication use for anxiety or depressive symptoms, perceived needs for care and socio-demographic data if they met DSM-IV criteria for generalized anxiety disorder, panic disorder, agoraphobia, social phobia or depression in the last 12 months; or if they reported a high level of anxiety or depression symptoms combined with any of the following: current medication use, an anxiety or depressive disorder diagnosis by a healthcare professional, or DSM-IV criteria for anxiety or depression in the past 24 months. A total of 1956 participants completed both parts of the phone/web survey and were positive cases for any CIDIS disorder(s) (generalized anxiety disorder, panic disorder, agoraphobia, social anxiety and/or major depression). For this study, we included only the subset of 289 participants aged between 18 and 65 years old meeting DSM-IV criteria for SAD according to the CIDIS.

### Variables

#### Indicators for detection, service utilization and treatment adequacy

Depending on the timeframe, there were two types of “detection” indicators. First, we collected data on the detection of SAD, major depression and other anxiety disorders based on participants reporting having been informed of the diagnosis by a healthcare professional in the previous 12 months. Secondly, the lifetime detection of any anxiety disorders as well as the detection of chronic physical disorders were based on participants reporting of a diagnosis by a physician.

Indicators for service utilization included: 1) hospitalization for at least one night for mental health reasons in the 12 months preceding the interview; and 2) consultation with at least one health professional for mental health reasons in the previous 12 months (including general practitioners, psychologists, social workers, counsellors or psychotherapists, nurses, psychiatrists, or other specialized physicians).

Treatment adequacy included pharmacotherapy adequacy, psychotherapy adequacy and overall adequacy, which were defined using indicators based on the Canadian CPGs [24] using available proxies. Our indicator of treatment adequacy for pharmacotherapy was defined as: 1) reporting using evidence-based medication for SAD with a level 2 or higher level of evidence [24] 2) at an adequate dosage, while 3) having attended 3 or more visits with a general practitioner or a psychiatrist in the previous 12 months. Our indicator of treatment adequacy for psychotherapy was defined as reporting all the following: 1) having attended at least 8 visits with the same healthcare professional (general practitioner, psychologist, psychiatrist, counsellor, psychotherapist or social worker); 2) having received at least 15 minutes of counselling or psychotherapy; 3) having attended psychotherapy including a probable CBT approach. The probable CBT approach indicator was determined by a set of questions asking the type of received psychological help. Participants were first asked if they received psychological help in the form of psychotherapy/counselling session with a professional (≥15 minutes) in the past 12 months. Then, they were asked if they had experienced cognitive behaviour therapy (i.e. learning how to change thoughts, behaviours and emotions). These indicators were chosen using the 2006 Canadian Anxiety Guidelines [24], which were the current guidelines at the time of the "Dialogue" study.

#### Individual-level patient characteristics

Individual characteristics were organized according to Andersen’s Behavioural Model of Health Care [17] which is a conceptual framework operationalizing three types of factors associated with the use of health services: predisposing factors refer to an individual’s socio-cultural characteristics; enabling factors facilitate or support the use of healthcare; and need factors indicate the evaluated or perceived need for healthcare [17]. Variable selection was made according to previous literature, potential clinical relevance and the appropriateness of deriving proxies from available self-reported data [8–16,25,26]. The included three predisposing factors were gender (male; female), age group (18–24; 25–45; 46–65) and education level (high school or less; college degree; university degree). The five enabling factors included perception of income (poor/very poor; sufficient; more than enough), marital status (single; married/living together; separated/divorced/widowed), private insurance coverage for complementary health services (yes; no), affiliation with a family physician (yes; no), occupation (working or studying full time: yes; no). The four need factors were detection of SAD (yes; no), detection of comorbid major depression (yes; no), detection of comorbid anxiety disorders (yes; no) and comorbid chronic physical illness(es) (yes; no).

### Statistical analysis

Participant characteristics, service utilization and treatment adequacy based on our indicators were described using frequencies and proportions. Variables with insufficient case numbers to satisfy statistical test postulates were either excluded from further analysis or categories were combined, if conceptually relevant. Analysis were carried out independently for the following adequacy indicators as dependent variables: 1) pharmacological treatment; 2) psychological treatment; and 3) pharmacological and/or psychological treatment. As the definition of adequate psychotherapy for SAD varies considerably between studies, we also ran a sensitivity analysis using ≥12 sessions as the criterion for the adequate number of sessions (instead of 8). All independent variables were kept in each logistic regression model to allow for direct model comparison between adequacy indicators.

For chronic comorbid illness, we included pain [27,28], pulmonary [27,29], circulatory [27,29,30] and gastro-intestinal disorder [27,31] on the account of the complex relation that has been established between anxiety and these illnesses. Other physical comorbidity variables were added to the model individually to rule out potential unknown associations, but none were retained as they did not improve the goodness of fit of the model.

Binary logistic regression assumes a dichotomic dependent variable, independent observations, and little or no multicollinearity among independent variables. Collinearity was assessed in two steps. First, we used the phi coefficient (for dichotomic variables) and Cramer’s V (for categorical variables) to estimate binary correlations. Secondly, we used the generalized variance inflation factor statistic to assess multicollinearity, with a threshold of 10. No assumption violation has been detected. Logistic regression was performed for each dependent variable using α = 0.05. All analysis were conducted with SPSS version 24. The generalized variance inflation factor has been obtained through R 3.4.1 using the stepVIF function included in the Pedometrics package [32].

Study participants provided written informed consent at the time of the initial contact in primary care clinics after a lay-interviewer carefully explained the study aims and ethical considerations. Lay-interviewers received initial training and ongoing supervision, and the capacity to consent was evaluated verbally by the interviewers (without specific psychological tests). The Dialogue Project study, including the recruitment process and consent procedure, received the approval of all regional research ethics committees (Agence de santé de services sociaux de Montréal; Centre de santé et de services sociaux de Chicoutimi, Sherbrooke, Gatineau, Laval, Saint Jérome, Jeanne-Mance, Lac-Saint-Jean-Est, Pointe-De-L’ile, Bordeaux-Cartierville-Saint-Laurent, Therese-De-Blainville, Pierre Boucher, Haut-Richelieu-Rouville, Baie des Chaleurs, La Pommeraie; Hospital Notre-Dame and Hospital Sacré-Coeur).

## Results

### Participant characteristics

The sample characteristics of the T0 and T1 samples have been presented in a previous article [20] and assessed for selection bias in a previous research report [33; in French]. Respondents at T0 were more likely to be women and younger than non-respondent. Compared to the non-respondent, in the T1 sample, there were fewer people aged 65 or more, fewer men, more people with a self-reported low or moderate mental health, more diagnosed physical and mental illness, more people using medication for anxiety or depression and more people who had consulted a psychologist. Table 1 reports the individual characteristics of the selected subsample of 289 individuals in the Dialogue project aged between 18 and 65 years old that met DSM-IV criteria for SAD according to the CIDIS in the past 12 months. The majority of our sample were females (73.0%). The mean age was 41 (SD: 12.5) years old and half of the sample was either working or studying full time (51.2%). A large proportion of individuals had private medication insurance coverage (64.4%) and insurance coverage for complementary health services (50.7%) such as chiropractic, physiotherapy or psychotherapy. In our sample, 8 out of 10 individuals reported having a family physician.

**Table 1:** Individual characteristic of participants (n = 289)^a^.

Characteristic	Frequency	%
Sex (female)	211	73.0
Age group, years		
*18–24*	34	11.8
*25–45*	134	46.4
*46–65*	121	41.9
Marital status		
* Married/living together*	139	48.1
* Separated/divorced/widowed*	54	18.7
* Single*	96	33.2
Education level		
* High school degree or less*	148	51.4
* Collegial degree*	81	28.1
* University degree*	59	20.5
Participant perception of income		
* Financially secure*	31	10.8
* Sufficient*	151	52.4
* Poor/very poor*	106	36.8
Private medication insurance coverage (Yes)	186	64.4
Supplementary insurance coverage for complementary health services (Yes)	145	50.7
Has a family physician (Yes)	237	82.0
Comorbid anxiety disorder in the last 12 months (GAD, panic disorder, agoraphobia) ^b^	205	70.9
* Generalized anxiety disorder*	117	40.5
* Agoraphobia*	138	47.8
* Panic disorder*	104	36.0
Comorbid major depression in the last 12 months (yes)^b^	186	64.4
Any other long-term mental health condition ^c^	16	5.5
Comorbid chronic physical illnesses ^c^		
* 0*	69	23.9
* 1*	72	24.9
* 2*	54	18.7
*3 or more*	94	32.5

^a^ Varying between 285 and 289

^b^ Based on the CIDIS assessment

^c^ Participant reported diagnosis by a physician

In our sample, 57.4% of participants reported detection of a comorbid anxiety disorder by a healthcare professional in the previous 12 months (at least one comorbid agoraphobia, panic disorder or generalized anxiety disorder) and 46.4% reported comorbid major depression. For only 28.4% of the sample, no anxio-depressive comorbidity were detected. Excluding mood and anxiety disorders, 5.5% of the sample reported having a long-term comorbid mental disorder diagnosed by a health care professional. The two most frequent amongst these other mental illnesses were attention deficit/hyperactivity disorder and personality disorders. On average, participants reported experiencing their first social anxiety symptoms at 21 years of age (SD: 10.9) as part of the CIDIS interview [23] when asked about the first time they experienced intense and irrational fear of social situations. At least three in four individuals reported being diagnosed by a physician with a comorbid chronic physical illness. The most prevalent chronic physical illnesses reported were back pain (25.3%), migraine (23.2%) and hypertension (18.0%).

### Service utilization

Table 2 shows descriptive data concerning mental health service utilization in our sample. Overall, 86.2% of the sample had consulted at least one professional for mental health reasons in the previous 12 months. Among service users, 85.1% reported having seen a general practitioner for a median of 4 (interquartile range: 5) visits. As for psychologists, 48.8% of individuals had seen one in the last 12 months with a median of 3 (interquartile range: 5.25) visits. Throughout their life, 63.0% of participants reported at least one anxiety disorder diagnosed by a physician. In our sample, among participants reporting at least one consultation with a health professional for mental health reasons in the past 12 months, one in four participants also reported SAD detection by a healthcare professional in the previous 12 months.

**Table 2:** Service utilization of individuals with SAD (n = 289).

INDICATOR	Frequency	%
Was hospitalized for at least one night for mental health reasons in the past 12 months (Yes)	33	11.4
Consulted at least one health professional for mental health reasons in the past 12 months *Among participants who have consulted*	249	86.2

*General practitioner*	211	85.1
* Psychologist*	121	48.8
*Social worker/counsellor/psychotherapist*	96	38.7
* Psychiatrist*	81	32.5
* Nurse*	70	28.3
* Other medical specialist*	33	13.3
Anxiety disorder(s) detected by a physician during the life course (Yes)	182	63.0
Detection of SAD by a healthcare professional in the past 12 months (Yes)	65	23.6
*Among those who have consulted at least one health professional for mental health reasons in the past 12 months*	61^a^	25.7

^a^ total n = 237

### Treatment adequacy

Table 3 presents our indicators of treatment adequacy. Irrespective of adequacy status, antidepressants were the most received treatment (43.0%) among those who reported receiving any psychotropic medication (57.1%). Regarding pharmacotherapy adequacy, 32.2% of the participants met our criteria for receiving an evidence-based medication for SAD at minimally adequate dosage plus at least three visits with a general practitioner or a psychiatrist.

**Table 3:** Treatment adequacy indicators of individuals with SAD (n = 289).

INDICATOR	Frequency	%
*Pharmacotherapy*		
Psychotropic medication in the last 12 months	165	57.1
*Among psychotropic users*,		
*SSRIs*	71	43.0
* Benzodiazepines*	61	37.0
* Antipsychotics*	34	20.6
* MAOIs*	13	7.9
* Anticonvulsants*	2	1.2
* TCA*	1	0.6
* Other*	71	43.0
Received an evidence-based SAD medication	136	47.1
Received an evidence-based SAD medication at an adequate dosage	117	40.5
Received an evidence-based SAD medication at an adequate dosage, plus at least 3 consultations with a general practitioner or psychiatrist^a^	93	32.2
Received an evidence-based SAD medication at an adequate dosage for at least six months plus at least 3 consultations with a general practitioner or psychiatrist	58	20.1
*Psychotherapy*		
Any form of psychotherapy or counselling	143	49.5
Any form of psychotherapy or counselling lasting ≥ 15 minutes	117	40.5
*Probable problem solving therapy approach*	99	86.1
*Probable CBT approach*	96	84.2
*Probable interpersonal psychotherapy approach*	65	59.6
Psychotherapy with ≥ 8 sessions with a same healthcare professional lasting ≥ 15 minutes	73	26.0
Psychotherapy, probable CBT approach and ≥ 8 sessions with a same healthcare professional lasting ≥ 15 minutes ^b^	58	20.6
Psychotherapy with ≥ 12 sessions with a same healthcare professional lasting ≥ 15 minutes	51	17.6
Psychotherapy, probable CBT approach and ≥ 12 sessions with a same healthcare professional lasting ≥ 15 minutes	44	15.2
*Pharmacotherapy and*/*or Psychotherapy*		
Received an evidence-based pharmacological treatment and/or psychotherapy	117	40.5

^a^Our indicators for potentially adequate pharmacotherapy

^b^Our indicators for potentially adequate psychotherapy

Approximately half of the participants reported previous participation in any counselling or psychotherapy session (49.5%). Regarding specific components of these sessions, most participants reported components of problem solving therapy (86.1%) or CBT (84.2%). However, only 20.6% of participants met our psychotherapy adequacy definition by attending 8 sessions including at least 15 minutes of counselling with a probable CBT approach, with the same healthcare professional. Overall, 40.5% of our sample received adequate pharmacotherapy or psychotherapy according to our indicators.

### Factors associated with our indicators of treatment adequacy

Table 4 presents results of bivariate and multivariate logistic regression analysis showing association between predisposing, enabling or need factors and treatment adequacy based on our indicators.

**Table 4:** Predisposing, enabling and need factors associated with our indicators of treatment adequacy.

	Pharmacotherapy (n = 263) ^a^		Psychotherapy (n = 255) ^b^		Overall (n = 263) ^c^	
	Bivariate association	Multivariate association	Bivariate association	Multivariate association	Bivariate association	Multivariate association
	p	OR (95% CI)	p	OR (95% CI)	p	OR (95% CI)
**Predisposing factors**						
Gender (female)	0.755	0.886 (0.438–1.795)	0.490	1.191 (0.531–2.667)	0.876	0.836 (0.425–1.644)
Age group						
*18–24*	0.528	0.715 (0.255–2.007)	0.672	0.835 (0.263–2.653)	0.444	0.700 (0.266–1.844)
*25-45(ref)*		1.000		1.000		1.000
*46–65*	0.761	0.685 (0.345–1.358)	0.817	0.693 (0.322–1.492)	0.642	**0.508 (0.260–0.993)**
Education						
*High school or less (ref)*		1.000		1.000		1.000
*College degree*	0.074	1.727 (0.855–3.488)	0.864	0.903 (0.393–2.072)	0.152	1.641 (0.823–3.274)
*University degree*	0.973	0.804 (0.340–1.904)	0.069	2.374 (0.964–5.844)	0.638	1.198 (0.538–2.667)
**Enabling factor**						
Working or studying full time (yes)	0.056	0.658 (0.340–1.275)	**0.018**	**0.446 (0.203–0.981)**	**0.009**	**0.523 (0.275–0.995)**
Perception of incomes						
*Poor/very poor*	0.924	0.765 (0.378–1.547)	0.334	1.535 (0.686–3.435)	0.342	1.084 (0.553–2.125)
*Sufficient (ref)*		1.000		1.000		1.000
*More than enough*	0.469	0.703 (0.245–2.017)	0.693	0.912 (0.283–2.943)	0.760	0.934 (0.349–2.495)
Marital status						
*Single (ref)*		1.000		1.000		1.000
*Married/living together*	0.959	1.159 (0.555–2.417)	0.248	0.757 (0.337–1.702)	0.747	1.106 (0.552–2.217)
*Separated/divorced/widowed*	0.471	1.291 (0.520–3.205)	0.450	0.797 (0.276–2.303)	0.912	0.896 (0.369–2.177)
Private insurance coverage for complementary health services (Yes)	**0.026**	1.864 (0.919–3.784)	0.114	2.071 (0.915–4.690)	**0.037**	1.841 (0.930–3.645)
Has a family physician (yes)	0.219	1.528 (0.625–3.735)	0.074	3.205 (0.984–10.445)	0.069	2.084 (0.880–4.938)
**Need factor**						
Detected SAD in the last 12 months (yes)	**0.017**	0.991 (0.479–2.052)	0.132	1.105 (0.486–2.513)	**0.001**	1.420 (0.696–2.899)
Detected major depression in the last 12 months (yes)	**0.000**	**5.492 (2.913–10.355)**	**<0.001**	**3.298 (1.599–6.801)**	**<0.001**	**4.651 (2.559–8.453)**
Detected comorbid anxiety disorder (s) in the last 12 months	**0.000**	**3.081 (1.545–6.145)**	**0.002**	2.180 (0.996–4.770)	**<0.001**	**2.957 (1.555–5.625)**
Detected comorbid chronic physical illness(es) (yes)^d^	0.631	1.105 (0.573–2.130)	0.861	0.837 (0.4000–1.750)	0.565	1.117 (0.592–2.111)

Bold = Significant (p<0.05)

^a^ Indicator defined as: adequate SAD medication at an adequate dosage, plus at least 3 consultations with a general practitioner or psychiatrist

^b^ Indicator defined as: psychotherapy with a probable CBT approach and ≥ 8 sessions of at least 15 minutes with the same healthcare professional

^c^ Indicator defined as: adequate pharmacological and/or adequate psychological treatment

^d^ Circulatory, pain, gastrointestinal or pulmonary chronic illness(es)

#### Pharmacotherapy adequacy

Neither predisposing nor enabling factors were significantly associated with pharmacotherapy adequacy based on our indicators. Regarding need factors, participants with comorbid major depression (OR = 5.492; CI 95% [2.913–10.335]) or comorbid anxiety disorders (OR = 3.081; CI 95% [1.545–6.145]) in the previous 12 months were more likely to receive an adequate pharmacological treatment according to our indicator based on Canadian clinical practice guidelines [24].

#### Psychotherapy adequacy

Regarding our indicator of psychotherapy adequacy, there was no association with selected predisposing factors in multivariate analysis. Among enabling factors, full-time working or studying was associated with a lower probability of psychotherapy adequacy (OR = 0.446; CI 95% [0.203–0.981]). Regarding need factors, comorbid major depression (OR: 3.298; CI 95% [1.599–6.801]) was associated with higher treatment adequacy. Sensitivity analysis using 12 for the minimal number of sessions as a criterion for adequate psychotherapy instead of 8 can be found in S1 Table. In sensitivity analysis, comorbid anxiety disorder(s) was also associated with higher treatment adequacy for psychotherapy (OR: 2.889; CI 95% [1.183–7.059]).

#### Overall adequacy

Factors positively associated with our indicators of adequate pharmacotherapy and/or psychotherapy were both need factors, namely the diagnosis of comorbid major depression (OR = 4.651; CI 95% [2.559–8.453]) and anxiety disorder(s) (OR = 2.957; CI 95% [1.555–5.625]) in the past 12 months by a healthcare professional. The predisposing factor of being between 46 and 65 years old (OR = 0.508; CI 95% [0.260–0.993]) and the enabling factor of working or studying full time (OR = 0.523; CI 95% [0.275–0.995]) were both associated with lower treatment adequacy. In the sensitivity analysis using ≥ 12 sessions instead of ≥ 8 as the minimum number of sessions for adequate psychotherapy (S1 Table), having private insurance covering supplementary health services was associated with higher overall treatment adequacy (OR = 2.116; CI 95% [1.055–4.246]).

## Discussion

Data from the Dialogue cross-sectional survey in primary care clinics allowed us to assess primary care mental health service utilization, to evaluate treatment adequacy based on our indicators for SAD and to determine which factors were associated with adequacy using Andersen’s Behavioural Model of Health Care [17]. Reported mental health service utilization was high in our sample, estimated at 86.2%. Previous studies have found that only a low proportion of individuals living with SAD seeks help for their condition [34–36]. It has been suggested that some individuals may not recognize having an emotional problem or that they could benefit from mental health counselling [36]. The high service utilization may be explained by our primary care sampling strategy and high levels of psychiatric comorbidity observed among participants with SAD. Recruitment in our study required consulting for oneself in primary care, and characteristics of respondents in contact with primary health care services may differ from SAD in the general population. Furthermore, participants, while not seeking help for their SAD, could seek help for comorbid anxiety or depressive disorders, which they may think requires more attention [36]. It has been shown that the presence of comorbid depression in individuals with anxiety disorders increases service use for mental health problems [37–39]. It is also possible that using a dichotomic indicator of any visit for mental health reasons may not be sensitive enough to capture the service utilization differences among anxiety disorders.

Even if the majority of patients with SAD reported mental health service use, the detection rate of SAD by a healthcare professional was low, estimated at 23.6%. As a comparison point, we found a detection rate of 52.5% for participants with generalized anxiety disorder in the Dialogue Project [18]. Low rates of SAD detection have also been reported elsewhere [40]. When there are multiple comorbidities, as in our sample, there is a possibility that only the primary diagnosis is mentioned to the patient by the healthcare professional. In that case, participants might still be treated simultaneously for multiple conditions as treatments show similarities across conditions, and undisclosed diagnosis does not necessarily mean lack of treatment, as shown by our treatment adequacy data. It is also possible that SAD symptoms are missed because of patients feeling uncomfortable or unwilling to talk about their emotional problems from fear of being stigmatized or negatively judged [41]. Health care professionals may also dismiss some symptoms, or attribute them to other conditions or personal attributes, such as shyness.

As expected, treatment adequacy based on our indicators was low to moderate for both pharmacotherapy and psychotherapy, respectively 32.2% and 20.6%. Overall, treatment adequacy rates based on our indicators were within range of what is found in the literature for SAD [8–10]. However, direct comparisons must be made with caution due to heterogeneous definitions of pharmacotherapy [8–10] and psychotherapy adequacy [8–10] as well as the difference between study settings [8,10]. For psychotherapy, while 49.9% reported having had any form of counselling or psychotherapy, the number of sessions required for our indicator of adequacy (≥ 8) has been achieved for less than half of the participants. The low treatment adequacy for psychotherapy based on our indicators may reflect the difficulty in the continuity with a sufficient number of sessions, rather than initial access to counselling or psychotherapy [42]. Interpretation of this finding should be tempered by the cross-dataset; some of the respondents may be currently undergoing psychotherapy. It is interesting to note that sensitivity analysis revealed that comorbid anxiety disorder(s) was associated with higher treatment adequacy for psychotherapy, indicating that more complex clinical presentation may necessitate more extensive treatment plan, as expected.

Comorbid major depression disorder was associated with treatment adequacy for SAD, which is consistent with other studies on anxiety disorders [9,11,12,18]. Comorbid anxiety disorders were also associated with treatment adequacy for SAD as found in the Canadian Community Health Survey [12]. Considering that comorbid anxiety disorders and/or major depression were the most significant factors associated with treatment adequacy, a probable explanation could be that comorbidity in our sample indirectly increased treatment adequacy because of shared adequate treatment options between SAD and other common mental disorders. In primary care, the detection rate of major depression have been shown to be higher than for anxiety disorders, including SAD [40]. Additionally, the absence of association between the detection of SAD and treatment adequacy in our regression models supports the role of comorbidities in treatment adequacy, although it was not possible to differentiate for which participants SAD was the primary versus a secondary mental health problem. Ruscio *et al*. (2008), using the National Comorbidity Survey Replication (NCS-R) data, found that 68.9% of individuals with lifetime SAD reported having received treatment for mental health problems while only 35.2% of individuals reported having received treatment specifically for their SAD [43].

Although this study provides useful insights on the treatment adequacy for SAD and its determinants, some limitations must be considered in the interpretation of the results. First, our treatment indicators are based on Canadian clinical guidelines and are believed to be adequate from a population perspective. However, in an evidence-based practice model for individual patients, they are only relevant when tailored for the patient needs through the clinical judgment of a healthcare professional, and in light of patient preferences. Accordingly, we did not expect complete agreement of practice with CPGs. Second, this study used data from a cross-sectional survey, with a possibility of social desirability and recall bias. The social desirability bias could have influenced the answer given by participants and implies that they may have answered according to what seemed more favourable to the interviewer. A recall bias could also have affected the accuracy of the information collected during the interview, particularly for our mental health service use indicators. A study by Drapeau *et al*. (2011) showed over-reporting of mental health service use on a 12-month timeframe [44]. Third, our dataset was collected in 2008–2009, and changes in mental health care policy and clinical practice could have lead to variations in rates of treatment adequacy for SAD. Access to psychotherapy in the public sector remains difficult in our health care context [45]. We expect that the treatment adequacy rates are still accurate for psychotherapy, but we anticipate that treatment adequacy rates for pharmacotherapy may have increased due to documented trends on antidepressant use [46]. We believe that data on treatment adequacy in our sample could be considered as a baseline to be taken into account in national quality improvement initiatives aiming at policy or practice change to improve treatment adequacy for anxiety disorders. There is a possibly that the high prevalence of mental health comorbidity in our sample is affected by a selection bias as, by comparison to the non-respondents at T1, respondents were more likely to be affected by mental illness. Selection bias is also a possible contributor to the high female:male ratio, although a high proportion of female participants was expected given the primary care sampling [47–49] and the higher prevalence of females among people living with SAD [1,2,50–56]. Finally, this study was not powered to specifically investigate the subsample of participants suffering from SAD; this could be reflected in the absence of an association for some variables with either pharmacotherapy or psychotherapy adequacy, while being significantly associated with overall adequacy, as it is the case with age.

## Conclusion

From a primary care standpoint, our results demonstrate that SAD is under-detected and under-treated. Both pharmacotherapy and psychotherapy adequacy were found to be low in this study for individuals living with SAD, and antidepressants remain the most prescribed treatment. Among factors associated with higher treatment adequacy, need factors, specifically the detection of comorbid anxiety or depressive disorders, show the strongest association with our treatment adequacy indicators. Since the detection of SAD itself was not found to be independently correlated with receiving treatment, one can wonder if treatment adequacy for SAD might be an artefact of shared treatment options with psychiatric comorbidities. In light of these results, efforts should be directed toward increasing the recognition and treatment of SAD to assure adequate treatment in hopes of fostering personal recovery.

## Supporting information

S1 Table Sensitivity analysis of the logistic regression models using ≥12 sessions as the minimum for psychotherapy adequacy. (DOCX)
