# Supplementary material for: Treatment adequacy for social anxiety disorder in primary care patients
Source: PLoS One. 2018 Nov 5;13(11):e0206357. doi: 10.1371/journal.pone.0206357 (PMC6218038; doi:10.1371/journal.pone.0206357)
Supplement: S1 Table — (DOCX) [file pone.0206357.s001.docx]

| **Table 4.** Sensitivity analysis of the logistic regression models using ≥12 sessions as the minimum for psychotherapy adequacy. | | | | | | |
| --- | --- | --- | --- | --- | --- | --- |
|  | Pharmacotherapy (n=263) ^a^ | | Psychotherapy (n=255) ^b^ | | Overall (n=263) ^c^ | |
|  | Bivariate association | Multivariate association | Bivariate  association | Multivariate association | Bivariate association | Multivariate association |
|  | p | OR (95% CI) | p | OR (95% CI) | p | OR (95% CI) |
| **Predisposing factors** |  |  |  |  |  |  |
| Gender (female) | 0.755 | 0.886 (0.438-1.795) | 0.061 | 1.978 (0.751-5.214) | 0.498 | 1.003 (0.503-1.997) |
| Age group |  |  |  |  |  |  |
| *18-24* | 0.528 | 0.715 (0.255-2.007) | 0.345 | 0.618 (0.169-2.266) | 0.317 | 0.640 (0.237-1.729) |
| *25-45(ref)* |  | 1.000 |  | 1.000 |  | 1.000 |
| *46-65* | 0.761 | 0.685 (0.345-1.358) | 0.166 | 0.497 (0.212-1.162) | 0.539 | **0.481 (0.244-0.946)** |
| Education |  |  |  |  |  |  |
| *High school or less (ref)* |  | 1.000 |  | 1.000 |  | 1.000 |
| *College degree* | 0.074 | 1.727 (0.855-3.488) | 0.332 | 1.326 (0.539-3.266) | 0.102 | 1.774 (0.883-3.566) |
| *University degree* | 0.973 | 0.804 (0.340-1.904) | **0.027** | 2.370 (0.861-6.520) | 0.842 | 1.041 (0.459-2.361) |
| **Enabling factor** |  |  |  |  |  |  |
| Working or studying full time (yes) | 0.056 | 0.658 (0.340-1.275) | **0.005** | **0.354 (0.154-0.814)** | **0.004** | **0.466 (0.243-0.894)** |
| Perception of incomes |  |  |  |  |  |  |
| *Poor/very poor* | 0.924 | 0.765 (0.378-1.547) | 0.874 | 1.044 (0.426-2.562) | 0.448 | 1.038 (0.524-2.056) |
| *Sufficient (ref)* |  | 1.000 |  | 1.000 |  | 1.000 |
| *More than enough* | 0.469 | 0.703 (0.245-2.017) | 0.689 | 0.890 (0.247-3.210) | 0.564 | 0.806 (0.293-2.217) |
| Marital status |  |  |  |  |  |  |
| *Single (ref)* |  | 1.000 |  | 1.000 |  | 1.000 |
| *Married/living together* | 0.959 | 1.159 (0.555-2.417) | 0.471 | 0.728 (0.300-1.764) | 0.962 | 1.218 (0.599-2.476) |
| *Separated/divorced/widowed* | 0.471 | 1.291 (0.520-3.205) | 0.456 | 0.756 (0.231-2.473) | 0.791 | 1.067 (0.434-2.619) |
| Private insurance coverage for complementary health services (Yes) | **0.026** | 1.864 (0.919-3.784) | 0.072 | 2.321 (0.940-5.730) | **0.019** | **2.116 (1.055-4.246)** |
| Has a family physician (yes) | 0.219 | 1.528 (0.625-3.735) | 0.153 | 2.162 (0.637-7.332) | 0.106 | 1.821 (0.761-4.359) |
| **Need factor** |  |  |  |  |  |  |
| Detected SAD in the last 12 months (yes) | **0.017** | 0.991 (0.479-2.052) | 0.373 | 0.989 (0.402-2.434) | **0.001** | 1.439 (0.701-2.954) |
| Detected major depression in the last 12 months (yes) | **<0.001** | **5.492 (2.913-10.355)** | **0.001** | **2.396 (1.089-5.271)** | **<0.001** | **4.693 (2.563-8.594)** |
| Detected comorbid anxiety disorder (s) in the last 12 months | **<0.001** | **3.081 (1.545-6.145)** | **0.002** | **2.889 (1.183-7.059)** | **<0.001** | **2.861 (1.488-5.500)** |
| Detected comorbid chronic physical illness(es) (yes)^d^ | 0.631 | 1.105 (0.573-2.130) | 0.864 | 0.973 (0.434-2.181) | 0.467 | 1.178 (0.619-2.241) |
| ^a^ Indicator defined as: adequate SAD medication at an adequate dosage, plus at least 3 consultations with a general practitioner or psychiatrist  ^b^ Indicator defined as: psychotherapy with a probable CBT approach and ≥ 12 sessions of at least 15 minutes with the same healthcare professional  ^c^ Indicator defined as: adequate pharmacological and/or adequate psychological treatment  ^d^ Circulatory, pain, gastrointestinal or pulmonary chronic illness(es) | | | | | | |
